# Real-world retention rates of biologics in patients with rheumatoid arthritis

**DOI:** 10.1038/s41598-023-48537-z

**Published:** 2023-12-01

**Authors:** Kenji Takami, Shigeyoshi Tsuji

**Affiliations:** 1 Department of Orthopaedic Surgery, Nippon Life Hospital, 2-1-54 Enokojima, Nishi-ku, Osaka, 550-0006 Japan; 2 Department of Rheumatology, Japan Community Healthcare Organization Osaka Hospital, Osaka, Japan

**Keywords:** Immunology, Rheumatology, Materials science

## Abstract

Although biologics have their own characteristics, there are no clear criteria for selecting them to treat the patients with rheumatoid arthritis. To assist in selecting biologics, we investigated the retention rates of biologics at our institution. We examined retention rates, and reasons for dropout for biologics in 393 cases and 605 prescriptions (of which 378 prescriptions were as naive) at our hospital since October 2003. Throughout the entire course of the study, etanercept (ETN) was the most frequently used biologic, followed by adalimumab (ADA) and tocilizumab (TCZ). When narrowed down to the later period from 2010, ETN was still the most used, followed by TCZ and abatacept (ABT). When the retention rates were compared in biologic naive patients, the retention rates were TCZ, ABT, ETN, certolizumab pegol (CZP), golimumab (GLM), infliximab (IFX), and ADA, in that order. The retention rates were better with the first use of each biologic. The main reasons for dropout were primary ineffectiveness, secondary ineffectiveness, and infection. ETN was the most used biologic in our hospital, with an increasing trend toward the use of non-TNF inhibitors. Retention rates were higher in non-TNF inhibitors.

## Introduction

Various biologics have been introduced in the treatment of rheumatoid arthritis (RA), and their therapeutic outcomes have been improving. In Japan, since the approval of infliximab in 2003, new biologics have been approved one after another in clinical use^1^.

Although each biologic has its own characteristics, there are no clear criteria for selecting the one to use, and the choice depends on the decision of the clinician. Since the oral surveillance test had been reported^2^, the use of biologics has been considered over JAK inhibitors, and it is expected that there will be increasing cases concerned about the choice of biologics.

To assist in the selection of biologics, we investigated the retention rates of biologics, excluding janus kinase (JAK) inhibitors and sarilumab, at our institution.

## Results

### Trends in biologics selection

**Figure 1:**
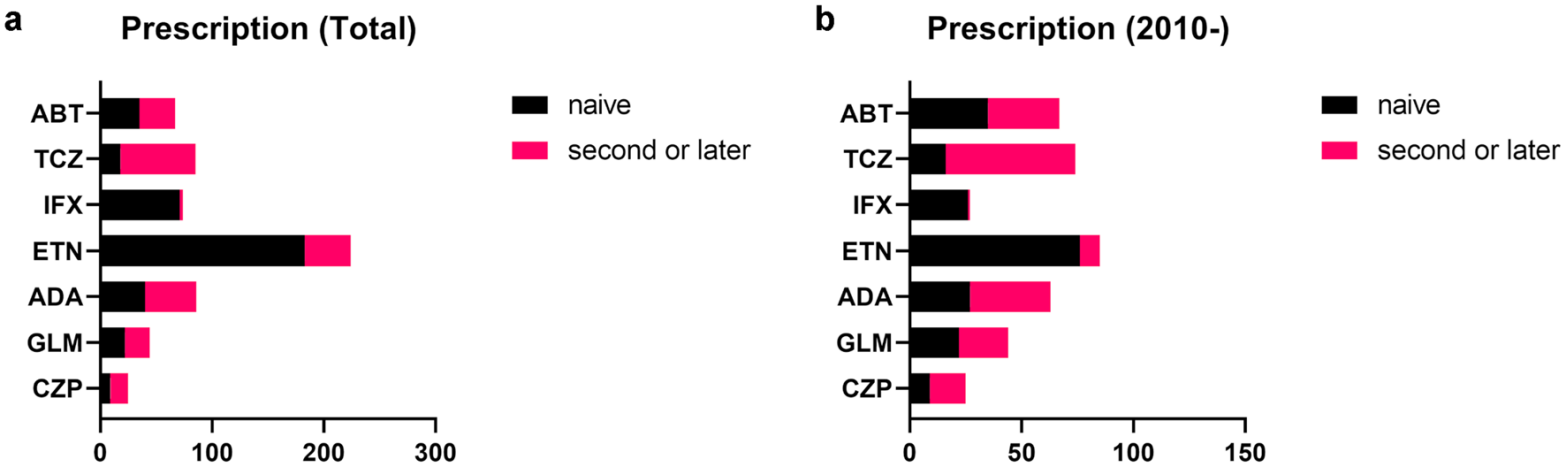
The prescription of biologics. (**a**) The prescription of biologics throughout the entire course of the study. (**b**) The prescription of biologics when narrowed down to the later period from 2010.

Throughout the entire course of the study, ETN was the most frequently used biologic, followed by ADA and TCZ (Fig. 1a).

When narrowed down to the later period from 2010, ETN was still the most used, followed by TCZ and ABT, indicating an increase in the use of non-tumor necrosis factor (TNF) inhibitors (Fig. 1b).

### Background factors

The background factors for each biologic are shown in Table 1. The background factors that differed among biologics were age, history of biologic use, and MTX usage. When comparing only in naïve patients, differences were observed in age and MTX usage, and blood data showed that KL6 tended to be higher in the non-TNF inhibitors group (Table 2).

**Table 1:** Baseline characteristics of patients in each group.

	ABT	TCZ	IFX	ETN	ADA	GLM	CZP	*p*-value
Cases (no.)	67	84	74	224	86	44	25	
Age (years)	69.1 ± 11.8	60.8 ± 15.3	51.6 ± 12.8	59.9 ± 16.1	61.9 ± 14.1	67.2 ± 9.75	61.2 ± 13.9	< 0.0001
Male rate (%)	23.9	20.2	32.4	16.5	18.6	18.2	20	0.1475
Body weight (kg)	54.8 ± 14.4	55.5 ± 11.5	58.4 ± 12.8	53.9 ± 11.5	54.4 ± 12.1	55.2 ± 13.5	55.6 ± 12.5	0.2771
BMI	22.7 ± 4.2	22.6 ± 3.9	22.5 ± 3.7	21.9 ± 3.7	22.1 ± 3.6	23.2 ± 4.8	22.5 ± 3.5	0.4376
Order of biologic use (no.)	2	2.3	1.1	1.2	1.7	2	2	< 0.0001
Biologic naïve patients (%)	52.2	21.4	95.9	81.7	46.5	50.0	36.0	< 0.0001
ACPA positivity (%)	89.6	97.6	90.5	88.2	87.1	88.6	92	0.3094
RF positivity (%)	82.1	86.9	82.2	78.5	75.6	81.8	76	0.5908
PSL usage (%)	61.2	61.9	71.8	63.8	60	65.9	48	0.3755
MTX usage (%)	70.1	67.9	98.6	72.9	82.6	93.2	80	< 0.0001
PSL dose (mg/day)	4.4 ± 2.7	5.2 ± 2.6	5.9 ± 2.8	5.4 ± 3.0	4.4 ± 2.2	4.5 ± 2.2	4.6 ± 2.0	0.0116
MTX dose (mg/week)	7.6 ± 3.7	8.5 ± 3.9	7.7 ± 2.6	6.9 ± 2.7	8.3 ± 3.9	7.6 ± 3.9	10.2 ± 3.8	< 0.0001

Data are shown as mean ± standard deviation, unless otherwise specified.

*BMI* body mass index, *ACPA* anti-cyclic citrullinated peptide antibody, *RF* rheumatoid factor, *PSL* prednisolone, *MTX* methotrexate, *ABT* abatacept, *TCZ* tocilizumab, *IFX* infliximab, *ETN* etanercept; *ADA* adalimumab, *GLM* golimumab, *CZP* certolizumab pegol.

**Table 2:** Baseline characteristics of patients of biologic naïve in each group.

	ABT	TCZ	IFX	ETN	ADA	GLM	CZP	*p*-value
Cases (no.)	35	18	71	183	40	22	9	
Age (years)	71.1 ± 10.2	63.1 ± 14.7	51.1 ± 12.8	60.8 ± 16.4	58.3 ± 12.5	66.2 ± 9.7	62.3 ± 7.3	< 0.0001
Male rate (%)	25.7	27.8	32.4	17.5	20	18.2	11.1	0.2211
Body weight (kg)	55.5 ± 12.7	56.1 ± 11.7	58.2 ± 11.7	53.9 ± 11.8	54.3 ± 10.0	53.5 ± 9.7	54.6 ± 9.2	0.2607
BMI	22.9 ± 4.2	22.5 ± 4.2	22.4 ± 3.4	22.0 ± 3.6	22.0 ± 3.0	22.4 ± 4.1	22.4 ± 3.5	0.9064
ACPA positivity (%)	88.6	100	90.1	87.2	82.1	86.4	88.9	0.6646
RF positivity (%)	80	100	81.4	76.4	72.5	77.3	77.8	0.3509
MMP3 (ng/ml)	312 ± 297.0	336 ± 361.5	362 ± 287.2	251 ± 251.1	238 ± 260.0	216 ± 228.7	290 ± 262.2	0.0811
PSL usage (%)	57	67	72	65	45	59	22	0.0157
MTX usage (%)	74	50	99	71	90	100	100	< 0.0001
PSL dose (mg/day)	5.1 ± 3.0	5.7 ± 2.4	6.0 ± 2.8	5.4 ± 3.0	4.2 ± 1.3	4.5 ± 2.8	4.5 ± 0.7	0.0035
MTX dose (mg/week)	7.5 ± 3.6	8.8 ± 3.6	7.9 ± 2.4	7.0 ± 2.9	8.2 ± 3.3	7.8 ± 3.5	10.9 ± 2.7	< 0.0001
CRP (mg/dl)	2.0 ± 1.8	3.2 ± 2.9	3.4 ± 3.9	2.4 ± 2.4	2.3 ± 2.7	2.6 ± 3.0	2.4 ± 2.8	0.1718
ESR (mm)	57.9 ± 25.9	69.4 ± 32.6	61.6 ± 35.8	64.2 ± 36.7	67.1 ± 37.7	67.5 ± 27.6	69.7 ± 41.4	0.8630
IgG (mg/dl)	1589.3 ± 380.6	1513.1 ± 427.9	1509.9 ± 383.1	1402.7 ± 375.6	1484.5 ± 347.8	1391.8 ± 310.1	1349.0 ± 335.9	0.2047
KL6 (U/ml)	387.9 ± 310.5	339.9 ± 194.6	208.2 ± 64.6	296.7 ± 251.0	259.6 ± 132.6	330.2 ± 219.1	311.2 ± 88.6	0.0147

Data are shown as mean ± standard deviation, unless otherwise specified.

*BMI* body mass index, *ACPA* anti-cyclic citrullinated peptide antibody, *RF* rheumatoid factor, *MMP3* matrix metalloprotainase-3, *PSL* prednisolone, *MTX* methotrexate, *CRP* C-reactive protein, *ESR* erythrocyte sedimentation rate, *IgG* immunoglobulin G, *KL6* sialylated carbohydrate antigen KL6, *ABT* abatacept, *TCZ* tocilizumab, *IFX* infliximab, *ETN* etanercept; *ADA* adalimumab, *GLM* golimumab, *CZP* certolizumab pegol.

**Figure 2:**
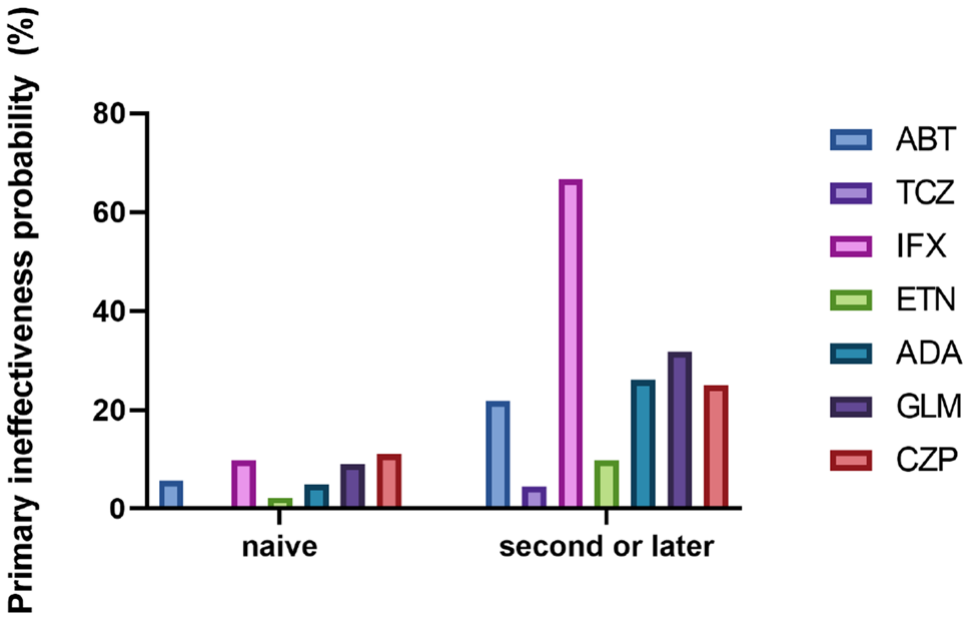
The rate of primary ineffectiveness of each biologic.

Each biologic tended to have an increased rate of primary ineffectiveness with second or later use (Fig. 2, Table 3). Conversely, no differences were observed in adverse effects (Table 3).

**Table 3:** Dropout due to primary ineffectiveness and adverse effect in patients who are naïve or not in each group.

	Naïve	Second or later	*p*-value
Primary ineffectiveness			
ABT (%)	6.1	28.0	0.0754
TCZ (%)	0.0	3.1	> 0.9999
IFX (%)	9.2	100.0	0.1838
ETN (%)	2.2	10.8	0.0391
ADA (%)	5.3	35.3	0.0090
GLM (%)	10.0	46.7	0.1324
CZP (%)	12.5	33.3	0.6206
Adverse effect			
ABT (%)	33.3	23.5	0.6322
TCZ (%)	50.0	60.0	> 0.9999
IFX (%)	37.5	37.0	> 0.9999
ETN (%)	35.7	32.4	0.7974
ADA (%)	12.5	17.1	> 0.9999
GLM (%)	50.0	25.0	0.3625
CZP (%)	0.0	18.2	> 0.9999

Data are shown as n (%), unless otherwise specified.

*ABT* abatacept, *TCZ* tocilizumab, *IFX* infliximab, *ETN* etanercept, *ADA* adalimumab, *GLM* golimumab, *CZP* certolizumab pegol.

### Retention rates

**Figure 3:**
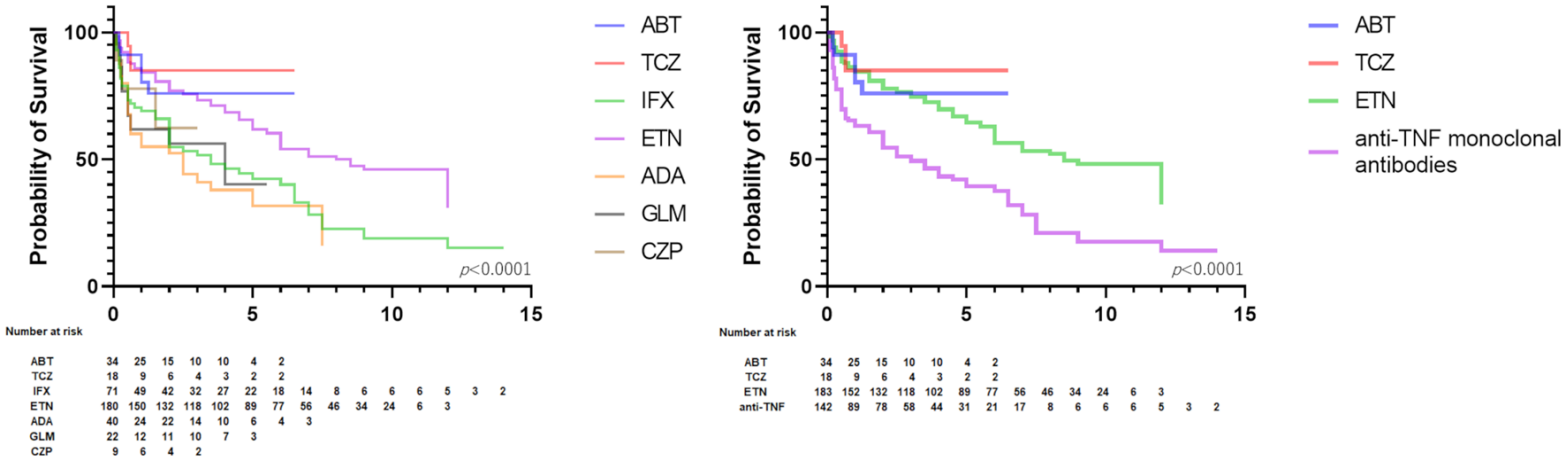
The retention rates of each biologic in naïve patients.

When the retention rates were compared only in naïve patients, the rates were TCZ, ABT, ETN, CZP, GLM, IFX, and ADA, in that order (Fig. 3). When TNF inhibitors other than ETN were grouped together and compared by group, TCZ, ABT, ETN, and anti-TNF monoclonal antibodies had the good retention rates, in that order (Fig. 3). Although the sample size is small, added the graph of retention rates adjusted for age, sex, BMI, ACPA and RF positivity, concomitant doses of PSL and MTX (Supplementary Fig. S1). The adjusted graph was compared to the unadjusted graph, but no change in trend was observed.

MTX use was significantly positively contributing to retention rates for ETN, ADA, and GLM (Supplementary Table S1).

**Figure 4:**
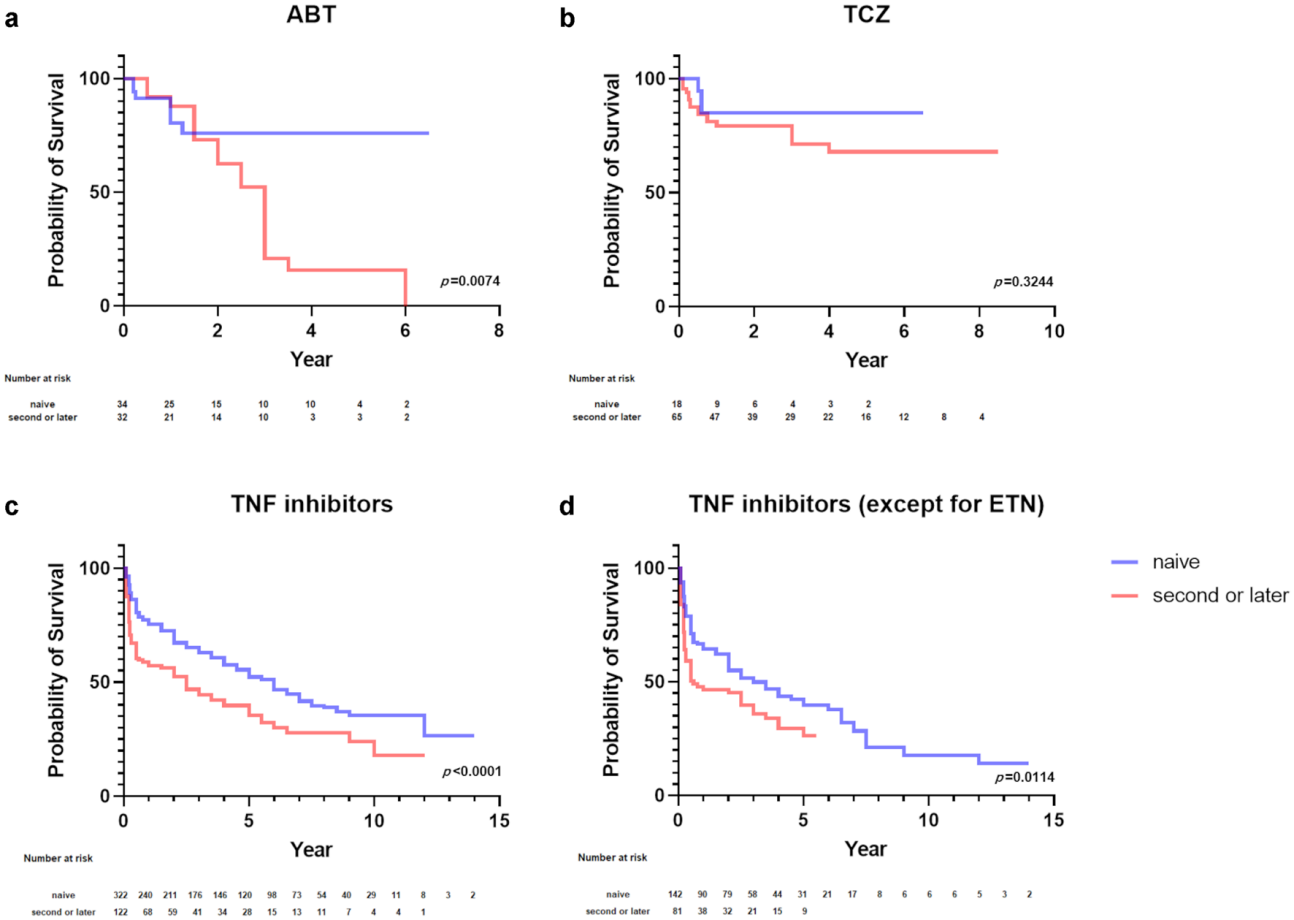
The difference in the retention rates of biologics between naïve patients and the other patients.

When the difference in the retention rates was compared between naïve patients and the other patients for each biologic, the rates were still better with the first use of each biologic. That was more pronounced for ABT and TNF inhibitors (Fig. 4a–c, Tables 4, 5, 6, Supplementary Figs. S2–S5, Supplementary Tables S2–S5). As ETN is Fc-fusion proteins with less immunogenicity, the retention rate of TNF inhibitors except for ETN was also evaluated (Fig. 4d). Overall, retention rates worsened, but still remained significantly better for use with naïve patients.

**Table 4:** Baseline characteristics of patients with biologic naïve or not.

ABT	Naïve	Second or later	*p*-value
Cases (no.)	35	32	
Age (years)	71.1 ± 10.2	67.0 ± 13.1	0.1612
Male rate (%)	25.7	21.9	0.7797
Body weight (kg)	55.5 ± 12.7	54.1 ± 16.3	0.7007
BMI	22.9 ± 4.2	22.6 ± 4.2	0.8204
ACPA positivity (%)	88.6	90.6	> 0.9999
RF positivity (%)	82.9	84.4	> 0.9999
MMP3 (ng/ml)	312.2 ± 297.0	244.4 ± 189.6	0.2763
PSL usage (%)	54.3	68.8	0.3160
MTX usage (%)	74.3	65.6	0.5938
PSL dose (mg/day)	5.1 ± 3.0	3.8 ± 2.3	0.1024
MTX dose (mg/week)	7.5 ± 3.6	7.8 ± 3.8	0.6708

Data are shown as mean ± standard deviation, unless otherwise specified.

*BMI* body mass index, *ACPA* anti-cyclic citrullinated peptide antibody, *RF* rheumatoid factor, *MMP3* matrix metalloprotainase-3, *PSL* prednisolone, *MTX* methotrexate, *ABT* abatacept.

**Table 5:** Baseline characteristics of patients with biologic naïve or not.

TCZ	Naïve	Second or later	*p*-value
Cases (no.)	18	66	
Age (years)	63.1 ± 14.7	60.2 ± 15.5	0.4717
Male rate (%)	27.8	18.2	0.5078
Body weight (kg)	56.1 ± 11.7	55.3 ± 11.6	0.7902
BMI	22.5 ± 4.2	22.6 ± 3.8	0.9510
ACPA positivity (%)	100	97	> 0.9999
RF positivity (%)	100	86.4	0.1941
MMP3 (ng/ml)	336.2 ± 361.6	325.5 ± 335.0	0.9061
PSL usage (%)	66.7	60.6	0.7862
MTX usage (%)	50	72.7	0.0890
PSL dose (mg/day)	5.7 ± 2.4	5.1 ± 2.7	0.4377
MTX dose (mg/week)	8.8 ± 3.6	8.5 ± 4.0	0.6741

Data are shown as mean ± standard deviation, unless otherwise specified.

*BMI* body mass index, *ACPA* anti-cyclic citrullinated peptide antibody, *RF* rheumatoid factor; *MMP3* matrix metalloprotainase-3, *PSL* prednisolone, *MTX* methotrexate, *TCZ* tocilizumab.

**Table 6:** Baseline characteristics of patients with biologic naïve or not.

TNF inhibitor	Naïve	Second or later	*p*-value
Cases (no.)	325	128	
Age (years)	58.8 ± 15.3	62.0 ± 14.5	0.0423
Male rate (%)	20.9	17.2	0.4331
Body weight (kg)	54.9 ± 11.4	55.3 ± 13.8	0.7427
BMI	22.1 ± 3.5	22.5 ± 4.4	0.3647
ACPA positivity (%)	87.2	92.2	0.1866
RF positivity (%)	78.6	85.2	0.1467
MMP3 (ng/ml)	267.8 ± 251.0	241.1 ± 268.3	0.3237
PSL usage (%)	62.3	64.8	0.6649
MTX usage (%)	82	79.7	0.5928
PSL dose (mg/day)	5.4 ± 2.8	4.8 ± 2.4	0.0274
MTX dose (mg/week)	7.6 ± 2.9	7.6 ± 3.9	0.5924

Data are shown as mean ± standard deviation, unless otherwise specified.

*BMI* body mass index, *ACPA* anti-cyclic citrullinated peptide antibody, *RF* rheumatoid factor, *MMP3* matrix metalloprotainase-3, *PSL* prednisolone, *MTX* methotrexate, *TNF* tumor necrosis factor.

**Figure 5:**
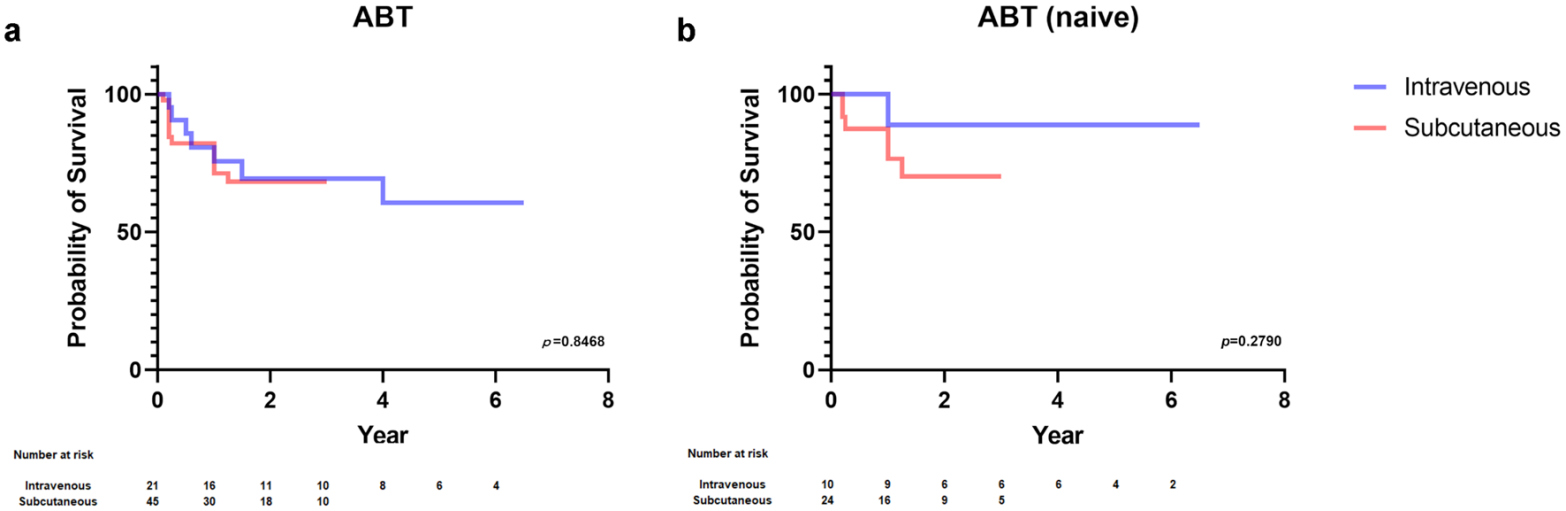
The difference in the retention rates of abatacept. (**a**) The difference in the retention rates of abatacept between intravenous and subcutaneous. (**b**) The difference in the retention rates of abatacept between intravenous and subcutaneous in naïve patients.

In addition, ABT was examined for the difference in the retention rates between intravenous and subcutaneous, however, no significant differences were found (Fig. 5a,b, Tables 7, 8).

**Table 7:** Baseline characteristics of patients using ABT through intravenous drip or subcutaneous injection.

ABT	Intraveous drip	Sc injection	*p*-value
Cases (no.)	21	46	
Age (years)	67.0 ± 11.6	70.1 ± 11.9	0.3278
Male rate (%)	19	26.1	0.7584
Body weight (kg)	53.7 ± 8.8	55.3 ± 16.5	0.6834
BMI	22.2 ± 2.2	23.0 ± 4.8	0.4934
Order of biologic use (no.)	2.1 ± 1.4	2.0 ± 1.4	0.61
ACPA positivity (%)	81	93.5	0.1932
RF positivity (%)	81	84.8	> 0.9999
MMP3 (ng/ml)	250.0 ± 251.1	293.1 ± 252.8	0.5202
PSL usage (%)	66.7	58.7	0.5976
MTX usage (%)	71.4	69.6	> 0.9999
PSL dose (mg/day)	4.5 ± 2.5	4.4 ± 2.8	0.7341
MTX dose (mg/week)	7.2 ± 2.4	7.8 ± 4.2	0.9135
csDMARDs usage (no.)	2.0 ± 0.9	1.8 ± 1.0	0.2645

Data are shown as mean ± standard deviation, unless otherwise specified.

*BMI* body mass index, *ACPA* anti-cyclic citrullinated peptide antibody, *RF* rheumatoid factor; *MMP3* matrix metalloprotainase-3, *PSL* prednisolone, *MTX* methotrexate, *csDMARDs* conventional synthetic disease-modifying antirheumatic drugs, *ABT* abatacept, *Sc* subcutaneos.

**Table 8:** Baseline characteristics of biologic naïve patients using ABT through intravenous drip or subcutaneous injection.

ABT	Intraveous drip	Sc injection	*p*-value
Cases (no.)	21	46	
Age (years)	73.6 ± 6.8	70.1 ± 11.3	0.3660
Male rate (%)	10.0	32.0	0.2346
Body weight (kg)	51.3 ± 6.9	57.2 ± 14.2	0.2213
BMI	21.5 ± 2.4	23.4 ± 4.7	0.2488
ACPA positivity (%)	70.0	96.0	0.0613
RF positivity (%)	80.0	84.0	> 0.9999
MMP3 (ng/ml)	251.4 ± 270.0	337.6 ± 309.4	0.4491
PSL usage (%)	60.0	52.0	0.7233
MTX usage (%)	80.0	72.0	> 0.9999
PSL dose (mg/day)	5.3 ± 2.5	5.1 ± 3.2	0.7303
MTX dose (mg/week)	6.3 ± 2.0	8.0 ± 4.1	0.4501
csDMARDs usage (no.)	1.8 ± 0.9	1.8 ± 1.1	0.8814
Disease duration (months)	14.0 ± 12.7	11.8 ± 15.1	0.6918
CRP (mg/dl)	1.7 ± 1.3	2.2 ± 2.0	0.4691
ESR (mm)	62.3 ± 27.7	56.2 ± 25.5	0.5337
KL6 (U/ml)	306.6 ± 141.1	419.7 ± 353.4	0.3626

Data are shown as mean ± standard deviation, unless otherwise specified.

*BMI* body mass index, *ACPA* anti-cyclic citrullinated peptide antibody, *RF* rheumatoid factor; *MMP3* matrix metalloprotainase-3, *PSL* prednisolone, *MTX* methotrexate, *csDMARDs* conventional synthetic disease-modifying antirheumatic drugs, *CRP* C-reactive protein, *ESR* erythrocyte sedimentation rate, *KL6* sialylated carbohydrate antigen KL6, *ABT* abatacept, *Sc* subcutaneous.

### Reasons for dropout

**Figure 6:**
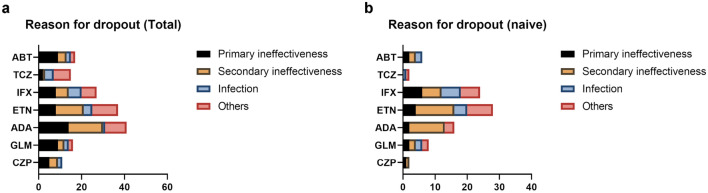
The reasons for dropout. (**a**) The reasons for dropout of each biologic. (**b**) The reasons for dropout of each biologic in naïve patients.

The reasons for dropout were examined for each biologic (Fig. 6a). The reasons for dropout were also examined only in naïve patients (Fig. 6b). In all cases, the main reasons were primary ineffectiveness, secondary ineffectiveness, and infection, and the dropout rate due to primary ineffectiveness was lower when each biologic was used in naïve patients than used in the second or later use patients. The dropout rate due to primary ineffectiveness was the lowest in TCZ group, although the difference was not significant (Table 9, Supplementary Table S6).

**Table 9:** Probability of dropout due to primary ineffectiveness.

	ABT	TCZ	TNFi	*p*-value
Total	9/17 (52.9)	2/15 (13.3)	43/132 (32.6)	0.0579
Naïve	2/6 (33.3)	0/2 (0.0)	15/78 (19.2)	0.5480

Data are shown as dropout due to primary ineffectiveness/total dropout (%).

*ABT* abatacept, *TCZ* tocilizumab, *TNFi* tumor necrosis factor inhibitors.

Conversely, the dropout rate due to infection was the highest in TCZ group, although the difference was not significant (Table 10, Supplementary Table S7).

**Table 10:** Probability of dropout due to infection.

	ABT	TCZ	TNFi	*p*-value
Total	2/17 (11.8)	4/15 (26.7)	16/132 (12.1)	0.2868
Naïve	2/6 (33.3)	1/2 (50.0)	12/78 (15.4)	0.2524

Data are shown as dropout due to infection/total dropout (%).

*ABT* abatacept, *TCZ* tocilizumab, *TNFi* tumor necrosis factor inhibitors.

The dropout due to infection is fully clarified in Supplementary Table S8.

## Discussion

Among biologics, ETN and TCZ were prescribed more frequently at our institution, and recently ABT has been increasingly prescribed. There were also differences in the background factors such as age, MTX usage, and KL6 levels. The tendency to use of ABT in older patients may reflect the finding that the risk of infection does not increase with age in postmarketing surveillance^3^. For the difference in MTX usage, ABT, TCZ, and ETN may be selected for patients who are unable to satisfactorily use MTX. Because TNF inhibitors, except for ETN which is relatively resistant to the emergence of anti-drug antibodies, have the high risk of immunogenicity when MTX cannot be used together^4,5^. Moreover, some reports indicate that there is no significant difference in the efficacy with and without MTX for ABT and TCZ^6,7^. TNF inhibitors are associated with a risk of exacerbation of interstitial lung disease^8,9^. The higher KL-6 in the ABT group may reflect the relatively safe use of ABT in patients at risk for interstitial pneumonia^8–10^.

TCZ had the best retention rate, followed by ABT and ETN like that reported previously^7,11–13^. Although the sample size is small, the retention rates adjusted for age, sex, BMI, ACPA and RF positivity, concomitant doses of PSL and MTX were unchanged from the unadjusted case. The reversal of the retention rates for ETN and TCZ compared to the previous report^14^ may be due to differences in sample size, age and percentage of males, as well as the authors’ affiliation. However, we believe that there is no contradiction in the tendency that the retention rates of ETN and TCZ are better than ADA and IFX. Except for ETN, TNF inhibitor users often experienced primary or secondary inefficacy as the cause of dropout unlike ABT, TCZ, and ETN, which may be one of the reasons for the differences in the retention rates in this study^5,15,16^. The emergence of anti-drug antibodies is one possible reason for the above^4,17–21^.

The difference in the retention rate between naïve patients and the other patients was examined. TNF inhibitors and ABT showed a significantly higher retention rate when used in naïve patients. Conversely in this study, TCZ had no difference in the retention rates between the groups as described in JAK inhibitors^22^, although previous reports have shown that a history of biologic use reduces the efficacy of not only ABT but TCZ^23–28^. In terms of retention rate, ABT is likely to be inadequate for difficult-to-treat patients who have not responded to previous biologics in this study.

Although changing of the mode of action is recommended when the first TNF inhibitor is ineffective^29–31^, it was suggested that TCZ may be better than ABT, as reported in the past^32,33^. Furthermore, we can keep TNF inhibitors rotation as a treatment option because the result of this study and the previous reports indicated the retention rate is not that bad^34–38^. Among TNF inhibitors, only CZP showed a significant difference in the retention rate depending on whether the patient had used biologics or not. However, further data accumulation is needed, given the low MTX usage and the small sample size in CZP group. Although there was no difference in the background factors and the retention rate between intravenous and subcutaneous treatment for ABT, the retention rate of intravenous tended to be higher in cases when ABT was used in naïve patients. IV infusion may be more likely to be effective because the dose can be adjusted according to body weight. However, the sample size is small and longer-term follow-up is needed.

The reasons for dropout were the same as in previous report^16^. Primary and secondary ineffectiveness and infection were the most common reasons for dropout. There was a trend toward more dropouts due to infection in TCZ as previously reported^39,40^. We cannot rule out the possibility that the present results are secondary to low ineffectiveness^16,41^.

Limitations include the single-center study, the change of the upper limit of MTX to 16 mg since 2011, the small number of cases and the variation of the number in each biologic group. Future studies should be conducted at multiple centers to increase sample size, reduce bias, and increase external validity. Furthermore, the background factors of patients such as smoking history, imaging test, and composite scores are not examined. The influence of low cost on the choice of TCZ cannot be denied. However, in Japan, the cost of biologics may have little influence on the choice due to its insurance system. In addition, although the preferences of the attending rheumatologists may have influenced the choice of biologics to some extent, we believe that this is the limitation that comes with real world data. On the other hand, it is also true that the retention rates that takes this into account would be useful in clinical practice. We believe that the various data in this study will be useful to clinicians who have the opportunity to use biologics in clinical practice.

In summary, we reviewed the details of the use of biologics for RA at our institution. The aging of the patients and the accompanying background factors were considered to influence the choice of biologics. Retention rates were higher for non-TNF inhibitors. As the options are now expanding with the release of sarilumab and JAK inhibitors, further studies including these drugs are needed in the future.

## Methods

We collected the data from RA patients who fulfilled the 1987 RA classification criteria of the American College of Rheumatology^42^ or the 2010 ACR/EULAR RA classification criteria^43^ at our hospital since October 2003.

In this study, we examined the consecutive patients who were treated with biologic disease-modifying antirheumatic drugs (bDMARDs), abatacept (ABT), adalimumab (ADA), certolizumab pegol (CZP), etanercept (ETN), golimumab (GLM), infliximab (IFX), and tocilizumab (TCZ) excluding biosimilar agents, with their demographic data, blood test data and reasons for dropout.

Demographic data include age, sex, body weight, body mass index (BMI), order of biologic use, rheumatoid factor (RF) and anti-cyclic citrullinated peptide antibody (ACPA) positivity, concomitant ratio and doses of prednisolone (PSL) and methotrexate (MTX), the number of concomitant conventional synthetic DMARDs (csDMARDs) use, and disease duration. Blood test data include matrix metalloprotainase-3 (MMP3), C-reactive protein (CRP), erythrocyte sedimentation rate (ESR), immunoglobulin G (IgG), and sialylated carbohydrate antigen KL6 (KL6). The retention rates of biologics were retrospectively evaluated as the duration until definitive treatment interruption. Reasons for dropout were classified into 4 major categories: (1) primary ineffectiveness; (2) secondary ineffectiveness; (3) infection; and (4) others.

Treatments were administered by the attending rheumatologists by the guidelines of the Japan College of Rheumatology^44–48^.

### Statistics

One-way analysis of variance, followed by a Tukey–Kramer post hoc test and Fisher’s exact probability test were used to analyze all values among the groups. Statistical analyses were performed using GraphPad Prism 9 (GraphPad, California, USA).

The graph of retention rates adjusted for age, sex, BMI, ACPA and RF positivity, concomitant doses of PSL and MTX was made using EZR (Saitama Medical Centre, Jichi Medical University, Saitama, Japan), a graphical user interface for R software (R Foundation for Statistical Computing, Vienna, Austria)^49^. The univariate and multivariate analysis of contributing factors to retention rates were also made using EZR.

### Study approval

All subjects gave written informed consent to participate in the study. Data were obtained in accordance with the Declaration of Helsinki. The study was approved by the Ethics Review Committee of Japan Community Healthcare Organization Osaka Hospital (reception number 2023-002).

### Supplementary Information

Supplementary Legends.

Supplementary Tables.

Supplementary Figure 1.

Supplementary Figure 2.

Supplementary Figure 3.

Supplementary Figure 4.

Supplementary Figure 5.

## Data Availability

The authors confirm that all relevant data are included in the article and/or its Supplementary Information files.
