# Supplementary material for: Real-world retention rates of biologics in patients with rheumatoid arthritis
Source: Sci Rep. 2023 Dec 1;13:21170. doi: 10.1038/s41598-023-48537-z (PMC10692158; doi:10.1038/s41598-023-48537-z)
Supplement: Supplementary file 1 — Supplementary Legends. [file 41598_2023_48537_MOESM1_ESM.docx]

Supplementary Figure 1. The retention rates of each biologic in naïve patients adjusted for age, sex, BMI, ACPA and RF positivity, concomitant doses of PSL and MTX.

Supplementary Figure 2. The difference in the retention rates of etanercept between naïve patients and the other patients.

Supplementary Figure 3. The difference in the retention rates of adalimumab between naïve patients and the other patients.

Supplementary Figure 4. The difference in the retention rates of golimumab between naïve patients and the other patients.

Supplementary Figure 5. The difference in the retention rates of certolizumab pegol between naïve patients and the other patients.
