# Supplementary figures and images for: Real-world retention rates of biologics in patients with rheumatoid arthritis
Source: Sci Rep. 2023 Dec 1;13:21170. doi: 10.1038/s41598-023-48537-z (PMC10692158; doi:10.1038/s41598-023-48537-z)

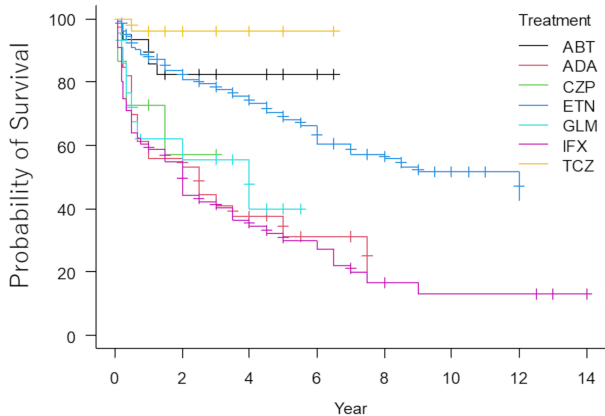

Number at risk

|     |     |     |    |    |    |    |   |   |
|-----|-----|-----|----|----|----|----|---|---|
| ABT | 33  | 15  | 5  | 2  | 0  | 0  | 0 | 0 |
| ADA | 39  | 22  | 10 | 3  | 0  | 0  | 0 | 0 |
| CZP | 9   | 4   | 0  | 0  | 0  | 0  | 0 | 0 |
| ETN | 169 | 124 | 95 | 73 | 44 | 23 | 3 | 0 |
| GLM | 22  | 11  | 7  | 0  | 0  | 0  | 0 | 0 |
| IFX | 65  | 36  | 22 | 13 | 6  | 4  | 4 | 2 |
| TCZ | 17  | 6   | 3  | 1  | 0  | 0  | 0 | 0 |

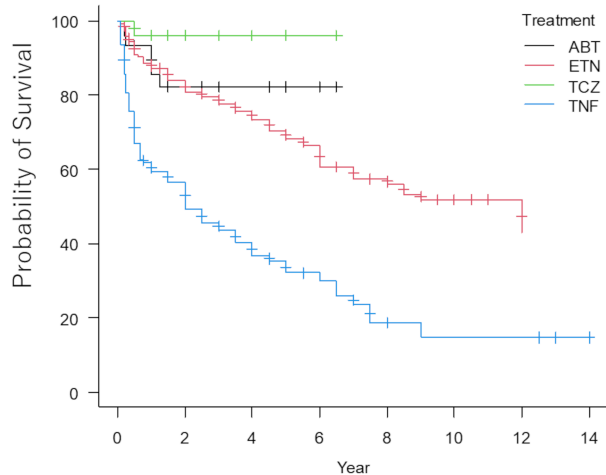

Number at risk

|     |     |     |    |    |    |    |   |   |
|-----|-----|-----|----|----|----|----|---|---|
| ABT | 33  | 15  | 5  | 2  | 0  | 0  | 0 | 0 |
| ETN | 169 | 124 | 95 | 73 | 44 | 23 | 3 | 0 |
| TCZ | 17  | 6   | 3  | 1  | 0  | 0  | 0 | 0 |
| TNF | 135 | 73  | 39 | 16 | 6  | 4  | 4 | 2 |

Supplement: Supplementary file 3 — Supplementary Figure 1. [file 41598_2023_48537_MOESM3_ESM.pdf]

# ETN

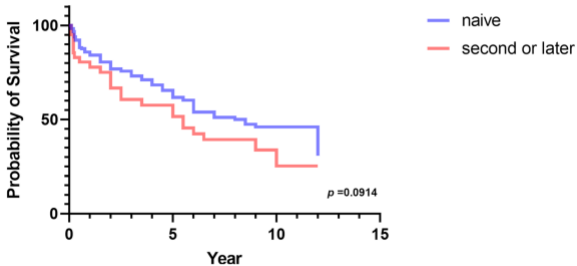

Number at risk

|                 |     |     |     |     |     |    |    |    |    |    |    |   |   |
|-----------------|-----|-----|-----|-----|-----|----|----|----|----|----|----|---|---|
| naive           | 180 | 150 | 132 | 118 | 102 | 89 | 77 | 56 | 46 | 34 | 24 | 6 | 3 |
| second or later | 41  | 30  | 27  | 22  | 20  | 19 | 15 | 13 | 11 | 7  | 4  | 4 | 1 |

Supplement: Supplementary file 4 — Supplementary Figure 2. [file 41598_2023_48537_MOESM4_ESM.pdf]

# ADA

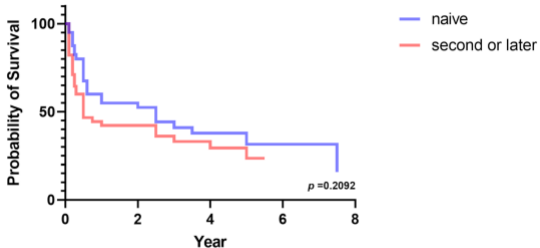

Number at risk

|                 |    |    |    |    |    |   |   |   |
|-----------------|----|----|----|----|----|---|---|---|
| naive           | 40 | 24 | 22 | 14 | 10 | 6 | 4 | 3 |
| second or later | 45 | 20 | 18 | 12 | 9  | 5 |   |   |

Supplement: Supplementary file 5 — Supplementary Figure 3. [file 41598_2023_48537_MOESM5_ESM.pdf]

# GLM

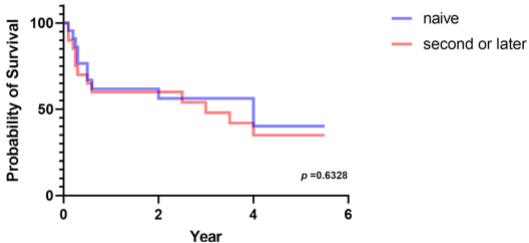

Number at risk

|                 |    |    |    |    |   |   |
|-----------------|----|----|----|----|---|---|
| naive           | 22 | 12 | 11 | 10 | 7 | 3 |
| second or later | 20 | 13 | 11 | 9  | 6 | 4 |

Supplement: Supplementary file 6 — Supplementary Figure 4. [file 41598_2023_48537_MOESM6_ESM.pdf]

# CZP

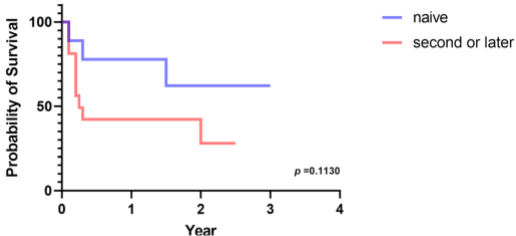

Number at risk

|                 |    |   |   |   |
|-----------------|----|---|---|---|
| naive           | 9  | 6 | 4 | 2 |
| second or later | 16 | 6 | 3 |   |

Supplement: Supplementary file 7 — Supplementary Figure 5. [file 41598_2023_48537_MOESM7_ESM.pdf]
