# Supplementary material for: Real-world retention rates of biologics in patients with rheumatoid arthritis
Source: Sci Rep. 2023 Dec 1;13:21170. doi: 10.1038/s41598-023-48537-z (PMC10692158; doi:10.1038/s41598-023-48537-z)
Supplement: Supplementary file 3 [file 41598_2023_48537_MOESM2_ESM.docx]

GLM (n=22)			
Age	1.064 (0.987 to 1.147)	0.1062	
Male	1.900 (0.501 to 7.213)	0.3456	
BMI	1.108 (0.969 to 1.267)	0.1347	
ACPA	1.000 (0.996 to 1.004)	0.9613	
RF	1.000 (0.996 to 1.003)	0.8215	
PSL	1.183 (0.984 to 1.423)	0.0744	1.199 (0.969 to 1.484)
MTX	0.808 (0.660 to 0.989)	0.0386	0.819 (0.672 to 1.000)
CZP (n=9)			
Age	0.871 (0.718 to 1.056)	0.1589	
Male	0.000 (0.000 to infinity)	0.9993	
BMI	0.488 (0.210 to 1.135)	0.0959	
ACPA	1.004 (0.999 to 1.009)	0.1389	
RF	1.001 (0.997 to 1.005)	0.6236	
PSL	1.307 (0.724 to 2.357)	0.3741	
MTX	0.897 (0.570 to 1.413)	0.6404	

BMI: body mass index; ACPA: anti-cyclic citrullinated peptide antibody; RF: rheumatoid factc prednisolone; MTX: methotrexate; ABT: abatacept; TCZ: tocilizumab; IFX: infliximab; ETN: etanercept; ADA: adalimumab; GLM: golimumab; CZP: certolizumab pegol

Cases (no.)	183	41
Age (years)	60.8±16.4	55.7±13.7
Male rate (%)	17.5	12.2
Body weight (kg)	53.9±11.8	54.3±10.2
BMI	22.0±3.6	21.7±4.0
ACPA positivity (%)	87.2	92.7
RF positivity (%)	78	90.2
MMP3 (ng/ml)	251.1±234.5	263.5±348.5
PSL usage (%)	65	58.5
MTX usage (%)	70.6	82.9
PSL dose (mg/day)	5.4±3.0	5.6±2.8
MTX dose (mg/week)	7.0±2.9	6.7±2.0

Data are shown as mean ± standard deviation, unless otherwise specified.
BMI: body mass index; ACPA: anti-cyclic citrullinated peptide antibody; RF: [illegible]
MMP3: matrix metalloprotainase-3; PSL: prednisolone; MTX: methotrexate; [illegible]

Cases (no.)	40	46
Age (years)	58.3±12.5	65.1±14.7
Male rate (%)	20	17.4
Body weight (kg)	54.3±10.0	54.6±13.7
BMI	22.0±3.0	22.2±4.1
ACPA positivity (%)	82.1	91.3
RF positivity (%)	77.5	82.6
MMP3 (ng/ml)	237.8±260.0	232.8±217.8
PSL usage (%)	45	67.4
MTX usage (%)	90	76.1
PSL dose (mg/day)	4.2±1.3	4.5±2.5
MTX dose (mg/week)	8.2±3.3	8.5±4.4

Data are shown as mean ± standard deviation, unless otherwise specified.
BMI: body mass index; ACPA: anti-cyclic citrullinated peptide antibody; RF: [illegible]
MMP3: matrix metalloprotainase-3; PSL: prednisolone; MTX: methotrexate; [illegible]

5

6

5
6

Supplementary Table 4. Baseline characteristics of patients with biologic naï

GLM	naïve	second or later
Cases (no.)	22	22
Age (years)	66.2±9.7	68.1±9.9
Male rate (%)	18.2	18.2
Body weight (kg)	53.5±9.7	56.8±16.5
BMI	22.4±4.1	24.0±5.4
ACPA positivity (%)	86.4	90.9
RF positivity (%)	77.3	86.4
MMP3 (ng/ml)	215.6±228.7	177.6±147.8
PSL usage (%)	59.1	72.7
MTX usage (%)	100	86.4
PSL dose (mg/day)	4.5±2.8	4.4±1.7
MTX dose (mg/week)	7.8±3.5	7.3±4.3

Data are shown as mean ± standard deviation, unless otherwise specified.
BMI: body mass index; ACPA: anti-cyclic citrullinated peptide antibody; RF: [illegible]
MMP3: matrix metalloprotainase-3; PSL: prednisolone; MTX: methotrexate; [illegible]

Supplementary Table 5. Baseline characteristics of patients with biologic naï

CZP	naïve	second or later
Cases (no.)	9	16
Age (years)	62.3±7.3	60.6±16.7
Male rate (%)	11.1	25
Body weight (kg)	54.6±9.2	56.2±14.2
BMI	22.4±3.5	22.6±3.5
ACPA positivity (%)	88.9	93.8
RF positivity (%)	77.8	75
MMP3 (ng/ml)	290.5±262.2	311.3±316.3
PSL usage (%)	22.2	62.5
MTX usage (%)	100	68.8
PSL dose (mg/day)	4.5±0.7	4.6±2.2
MTX dose (mg/week)	10.9±2.7	9.6±4.6

Data are shown as mean ± standard deviation, unless otherwise specified.
BMI: body mass index; ACPA: anti-cyclic citrullinated peptide antibody; RF: [illegible]
MMP3: matrix metalloprotainase-3; PSL: prednisolone; MTX: methotrexate; [illegible]
pegol

Supplementary Table 6. Probability of dropout due to primary ineffectiveness

	ABT	TCZ	IFX	ETN	ADA	GLM	CZP
total	9/17 (52.9)	2/15 (13.3)	7/27 (25.9)	8/37 (21.6)	14/41 (34.1)	9/16 (56.3)	5/11 (45.5)
naïve	2/6 (33.3)	0/2 (0.0)	6/24 (25.0)	4/28 (14.3)	2/16 (12.5)	2/8 (25.0)	1/2 (50.0)

Data are shown as dropout due to primary ineffectiveness / total dropout (%).
ABT: abatacept; TCZ: tocilizumab; IFX: infliximab; ETN: etanercept; ADA: adalimumab; GLM: golimumab; CZP: certolizu

Supplementary Table 7. Probability of dropout due to infection

	ABT	TCZ	IFX	ETN	ADA	GLM	CZP
total	2/17 (11.8)	4/15 (26.7)	7/27 (25.9)	4/37 (10.8)	1/41 (2.4)	2/16 (12.5)	2/11 (18.2)
naïve	2/6 (33.3)	1/2 (50.0)	6/24 (25.0)	4/28 (14.3)	0/16 (0.0)	2/8 (25.0)	0/2 (0.0)

Data are shown as dropout due to infection / total dropout (%).
ABT: abatacept; TCZ: tocilizumab; IFX: infliximab; ETN: etanercept; ADA: adalimumab; GLM: golimumab; CZP: certolizu

Supplementary Table 8. Infection in each group

ABT	TCZ	TNFi
pneumonia<br>bronchitis	pneumonia (2)<br>PCP<br>sepsis	pneumonia (10)<br>PCP (2)<br>pulmonary tuberculos[illegible]<br>appendicitis<br>cholesystitis<br>cholangitis

The number is listed in parentheses. N=1 if not stated.
ABT: abatacept; TCZ: tocilizumab; TNFi: tumor necros[illegible]
inhibitors; PCP: pneumocystis pneumounia [illegible]
